# Upper Rectal Cancer: To Irradiate or Not?

**DOI:** 10.7759/cureus.72973

**Published:** 2024-11-04

**Authors:** Basel Darawsha, Asaf Harbi, Myroslav Lutsky, Roi Abramov, Hayim Gilshtein

**Affiliations:** 1 General Surgery, Rambam Medical Center, Haifa, ISR; 2 Oncology, Rambam Medical Center, Haifa, ISR

**Keywords:** adenocarcinoma, anterior resection, low anterior resection syndrome, neoadjuvant chemoradiation, upper third rectal cancer

## Abstract

Introduction

This study examines the ongoing debate surrounding treatment strategies for upper rectal cancer. While neoadjuvant chemoradiotherapy (NCRT) is well established for mid and low rectal cancers, its efficacy for upper rectal cancers remains contentious. The objective of this study was to evaluate the safety, clinical outcomes, and oncologic results of chemoradiation in upper rectal cancer.

Methods

A retrospective cohort study was conducted at Rambam Health Care Hospital in Haifa, Israel, involving patients aged 18 and older diagnosed with locally advanced upper rectal cancer, defined as tumors located 11 to 15 cm from the anal verge, between 2013 and 2022. Patients were categorized into two groups: those who received NCRT prior to surgery and those who underwent surgery directly. The primary outcome measured was the incidence of postoperative complications, while secondary outcomes included mortality rates and the occurrence of local or distant recurrence.

Results

A total of 31 patients were included in the study, with 18 in the NCRT group and 13 in the surgery-first group. The two groups were comparable in terms of demographics and initial staging. The NCRT group exhibited a higher incidence of postoperative complications (66.7% vs. 38.5%), although this difference was not statistically significant. There were no significant differences in mortality rates or local recurrence between the groups. However, the NCRT group had a significantly higher incidence of low anterior resection syndrome (LARS) (27.8% vs. 0%).

Discussion

The findings suggest that NCRT does not enhance local control in upper rectal cancer compared to surgery alone. The increased incidence of LARS in the NCRT group underscores potential adverse effects associated with this treatment. These results are consistent with other studies that challenge the benefits of NCRT for upper rectal cancers, indicating a need for cautious interpretation and further research through larger, prospective studies.

Conclusions

NCRT for upper rectal cancer does not significantly improve local control and is associated with higher rates of LARS. Future studies should aim to optimize treatment protocols to balance efficacy and quality of life for patients with upper rectal cancer.

## Introduction

In the United States, approximately 46,000 new cases of rectal cancer are diagnosed annually [1]. The treatment of rectal cancer has undergone significant advancements in recent decades, with R0 resection remaining the cornerstone of management. Surgery alone is the preferred approach for early T1/2 lymph-negative tumors [2]; however, the decision-making process for locally advanced T3/4 rectal cancer is more complex. Treatment strategies for these patients typically involve considering neoadjuvant therapy. Various protocols for neoadjuvant chemoradiotherapy (NCRT) have been developed [3], aiming to reduce local recurrence and facilitate sphincter-sparing surgery for lower cancers, with recent trends toward organ preservation [4].

Tumor height plays a critical role in determining the management of rectal cancer. NCRT is well established for mid and low rectal cancers [5,6], but the optimal treatment for tumors located in the upper rectum remains a topic of debate. Achieving margins for higher lesions is relatively straightforward, and surgery for these tumors is generally considered less challenging, resulting in more favorable functional outcomes and lower ostomy rates. Furthermore, there is hesitance to administer radiation for upper rectal tumors due to potential toxic effects on adjacent structures, such as the bladder and small bowel.

In many centers, a surgery-first approach for resectable high rectal cancer is the mainstay of treatment. Local recurrence rates following anterior resection with partial mesorectal excision without neoadjuvant treatment have been reported to range from 4% to 8% in expert centers [7-9]. Conversely, some advocate for the widespread implementation of radiation therapy. The National Comprehensive Cancer Network guidelines recommend preoperative chemoradiotherapy for all stage II and III rectal cancers, irrespective of tumor location. Similarly, the American Society of Colon and Rectal Surgeons supports either preoperative or postoperative chemoradiotherapy for stage II and III upper rectal cancers [10].

This study aimed to examine the safety, clinical outcomes, and oncologic results of chemoradiation in upper rectal cancer.

## Materials and methods

This study utilized a retrospective cohort design, focusing on patients diagnosed with upper rectal cancer between 2013 and 2022. The research was conducted at Rambam Health Care Hospital, a tertiary medical center in Haifa, Israel. Institutional Review Board (IRB) approval was obtained prior to the study.

Study population

All patients aged 18 years and older diagnosed with locally advanced upper rectal cancer between 2013 and 2022 were included in the study. Upper rectal cancer was defined as tumors located 11 to 15 cm from the anal verge. After undergoing local and systemic staging, patients were presented to a multidisciplinary tumor board for treatment decision-making. The study group received neoadjuvant chemoradiation therapy, while the control group proceeded directly to surgery without neoadjuvant treatment. All patients received either continuous intravenous 5-fluorouracil or oral capecitabine at a dosage of 825 mg/m² twice daily (BID) weekly. A total dose of 45 Gy was delivered to the pelvis in five weekly sessions over five weeks, with 50 Gy targeted to the tumor. The boost volume included the tumor with an extension of 1 to 1.5 cm to account for potential tumor displacement.

Outcomes

The primary outcome measure was the incidence of postoperative complications. Secondary outcomes included mortality and the rates of local or distal recurrence.

Statistical analysis

Statistical analyses were conducted using IBM SPSS Statistics for Windows, Version 22.0 (Released 2013; IBM Corp., Armonk, NY, USA), with a significance level set at p < 0.05. Categorical variables were analyzed using the chi-square test, while continuous variables with a normal distribution were assessed using t-test. For continuous variables that did not follow a normal distribution, the Mann-Whitney test was employed.

## Results

During the study period, 31 patients were diagnosed with locally advanced upper rectal cancer. Among these patients, 18 (58%, Group 1) underwent NCRT prior to surgery, while the remaining 13 patients (42%, Group 2) underwent upfront surgery. The two groups were well-matched regarding age, gender, and BMI (Table 1).

**Table 1:** Comparison of baseline characteristics and staging between neoadjuvant and control groups Categorical variables were analyzed using the chi-square test, while continuous variables with a normal distribution were assessed using t-test.

Variable	Neoadjuvant (n = 18)	Control (n = 13)	Value	Df	p-value
General characteristics					
Age – years (SD)	57.2 (15.35)	63.7 (12.37)	-1.256	29	0.22
Sex – male (%)	10 (55.6%)	6 (46.2%)	0.267	1	0.6
Weight – kg (SD)	75.2 (17.9)	73.2 (10.31)	0.39	27.87	0.7
BMI – kg/m² (SD)	27.28 (4.85)	26.9 (2.57)	0.261	27	0.79
Staging (%)			4.59	2	0.1
T2 (%)	1 (7.7%)	0 (0%)			
T3 (%)	10 (76.9%)	18 (100%)			
T4 (%)	2 (15.4%)	0 (0%)			
Lymph node – N (%)			1.146	2	0.56
N0 (%)	8 (44.4%)	8 (61.5%)			
N1 (%)	9 (50%)	4 (30.8%)			
N2 (%)	1 (5.6%)	1 (7.7%)			

The comparison of initial pathological staging between the two groups revealed no significant differences. When examining the distribution of pathological staging across all patients, 45% were categorized as having a pathological stage of 3b. Subgroup analysis showed no significant difference between Group 1 (50%) and Group 2 (38.5%), with a p-value of 0.28. Regarding pre-surgery staging, all patients underwent CT for systemic staging.

Postoperative complications were classified into two categories: early complications, defined as those occurring within 30 days post-surgery, and late complications, which manifested more than one month after the surgical procedure. In Group 1, a total of 12 patients (66.7%) experienced either early or late complications, compared to five patients (38.5%) in the control group. However, this observed difference did not reach statistical significance, with a p-value of 0.11. In terms of mortality, no notable difference was found between the groups. Within Group 1, only one patient (5.6%) died after a postoperative period of four years, with the cause unrelated to the primary disease. None of the patients in the control group died during the follow-up period (Table 2).

**Table 2:** Postoperative outcomes and follow-up comparison between neoadjuvant and control groups in upper rectal cancer patients Categorical variables were analyzed using the chi-square test, while continuous variables with a normal distribution were assessed using t-test. LARS, low anterior resection syndrome

Variable	Neoadjuvant (n = 18)	Control (n = 13)	Value	Df	p-value
Death (%)	1 (5.6%)	0 (0%)	0.746	1	0.38
Follow-up – years (SD)	4.2 (2)	6.9 (4)	-2.06	16.3	0.056
Adjuvant therapy (%)	1 (5.6%)	7 (53.8%)	9.19	1	0.002
LARS	5 (27.8%)	0 (0%)	4.3	1	0.038
Overall complications (%)	12 (66.7%)	5 (38.5%)	2.42	1	0.12

The diagnosis of low anterior resection syndrome (LARS) was made by either the surgeon or the oncologist and subsequently confirmed by a gastrointestinal pelvic floor specialist. In the study group, five patients (27.8%) experienced LARS, while none of the patients in the control group who did not receive neoadjuvant radiation were affected.

## Discussion

The management of upper rectal cancer is controversial, with conflicting evidence from various studies. Our inquiry into the impact of NCRT on upper rectal cancer aimed to contribute to this ongoing debate. The Dutch TME trial [11] showed that neoadjuvant therapy did not significantly improve the local recurrence rate for patients with upper rectal cancer. Another pivotal study contributing to this understanding is the Swedish Rectal Cancer Trial [12], which indicated a statistically significant decrease in local recurrence rates for patients with low and medium rectal cancer who underwent neoadjuvant therapy. However, among the higher rectal cancer group, consisting of 143 patients, no significant decrease in local recurrence rates was observed between those who underwent a combination of radiotherapy and surgery and those who underwent surgery alone. Our study reinforces this conclusion, as there was no significant difference between the two groups regarding local recurrence.

Taking an opposing viewpoint, a study by Rades et al. [13] presented an alternative perspective. This study involved matching 52 patients with upper rectal cancer (defined as 10.1 cm or greater from the anal verge) to 208 patients treated with surgery alone. The neoadjuvant group displayed a trend toward better overall survival. The MRC CR07 and NCIC-CTG C016 study [14], a randomized controlled trial conducted across 80 centers in four countries, provided insights that differ from the Swedish trial [12]. It showed benefits for preoperative radiotherapy over selective postoperative chemoradiotherapy in high rectal cancer. The subgroup analysis of this study indicated an improved three-year local recurrence for neoadjuvant therapy (1.2%) compared to 6.2% in the control arm, emphasizing the potential efficacy of neoadjuvant approaches in decreasing local recurrence risks in high rectal cancer.

Examining the safety and potential postoperative complications of NCRT for upper rectal cancer, especially considering the proximity of the small intestine in the Douglas pouch to the radiation site, became a crucial aspect. The analysis of overall postoperative complications, encompassing late and early occurrences, revealed no statistically significant disparities between the two study groups. However, it is important to acknowledge that the study group had a shorter follow-up period. In terms of long-term complication rates, the study group manifested a 44.4% incidence, which included events of ileitis, small bowel obstruction, and LARS. In contrast, only one patient in the control group experienced a long-term complication, specifically a ventral hernia (7.7%, p-value = 0.026).

Surgical site infections are a notable postoperative complication in rectal surgery. These infections can lead to significant healthcare consequences, including extended hospital stays and increased rates of readmission, thereby impacting both patient recovery and healthcare resources [15].

Regarding mortality, one patient in the study group died during the follow-up period, with the cause unrelated to tumor recurrence. Additionally, one patient in the study group was diagnosed with distant metastasis during the follow-up period.

In concordance with the results of other studies [13,14], our study suggests that implementing neoadjuvant therapy maintains non-inferiority in preventing local recurrences. Neoadjuvant therapy may offer potential benefits such as tumor downstaging and improved R0 resection rates [4]. However, these potential benefits must be carefully weighed against the risks of radiation-induced toxicity and long-term complications such as LARS, which was observed more frequently in our neoadjuvant group. Notably, in the study group, five patients (27.8%) developed LARS, significantly higher than in the control group (p-value = 0.038). This finding aligns with the well-described impact of neoadjuvant therapy on LARS development.

Our study has several limitations, primarily attributable to its retrospective design. Additionally, there is a lack of consistency in the imaging modality performed for staging, specifically MRI. Another major limitation is the small sample size, which makes it challenging to draw definitive conclusions about recurrence and complication rates. This highlights the need for larger, more comprehensive studies.

The anatomic definition of the upper rectum based solely on distance from the anal verge is problematic and not entirely accurate. In our study, we described upper rectal cancer as tumors located 11-15 cm from the anal verge. This definition may not accurately reflect the tumor's relationship to the peritoneal reflection, which can vary significantly among patients. It has been described that gender significantly affects rectal anatomy, as the upper rectum in males is typically longer than in females [16]. Other factors influencing this anatomical variation include BMI and age [17].

## Conclusions

Our study did not find an improvement in local control with the administration of neoadjuvant chemoradiation for upper rectal cancer. Conversely, it did not increase short-term complications but was associated with a higher occurrence of LARS. These findings should be interpreted with caution, highlighting the need for larger studies, ideally with a multicenter prospective design, to further elucidate these outcomes.
